# Syphilitic Disease of the Nose

**Published:** 1892-09

**Authors:** C. G. Darling


### Syphilitic Disease of the Nose.

BY C. G. DARLING, M.D.

Clinical Lecturer on Oral Pathology and Surgery in the College of Dental Surgery of the University of Michigan.

A specific catarrh may begin in direct contamination of the nasal mucous membrane with syphilitic matter carried to it by means of instruments, or the uncleanly habit of picking the nose. It may be present in the secondary stage, while the nose is a common seat of tertiary lesions; it may also be the result of heredity.

When the initial sore appears in the nose there is swelling, pain, difficulty of respiration and fever. The eruption on the mucous membrane of the nose may be much the same as on the skin, or the appearance may be greatly modified by irritating secretions. Papular elevations rapidly lose their epithelium by having it degenerate into muco pus. This leaves the membrane a secreting surface of ashy color. When left to pursue their own course they spread, become cup-shaped and secrete large quantities of yellow pus. They also present a red border indicating a marked degree of congestion and favoring abundant secretion.

In the tertiary stage nodes are formed and the pressure of this adventitious tissue on the bloodvessels cuts off the blood supply, causing ulceration. This ulceration may terminate in resolution if treatment is begun early and little destruction of tissue result, or there may be marked cicatritial contraction, on account of the formation of connective tissue. When the underlying tissue or bone becomes involved it terminates in necrosis. The septum and the ethmoid bones are more commonly destroyed by ulceration than any other bones in the body.

The secondary lesions are announced by a mild coryza which gradually increases in intensity until it reaches the stage of purulent exudation. This discharge may undergo many changes but has a peculiar fetid odor, peculiar to syphilis.

Ulceration in the tertiary stage is most formidable. It first appears as a local swelling of variable size, gradually changing to a deep and ragged ulcer surrounded by a red areola. A greenish-yellow discharge covers the ulcer. The discharge may be streaked with blood, or carry with it shreds of necrosed tissue. Sometimes the secretion dries rapidly, forming hard crusts which rapidly decompose, producing a very offensive odor. The disease soon spreads to the underlying cartilage or bone and rapidly destroys it. The septum is first involved. The destruction of these bones of the nose causes the deformity to that part of the face so commonly seen in syphilitics.

When the turbinated bones are involved small pieces of bone may come away in the discharge, or may require removal. When the mucous membrane and the deeper parts begin to slough the odor is intolerable. Necrosis following gummatous affection of bone does not differ materially from other forms of necrosis. There may be new gummatous deposits about the edges of the diseased bone where the parts may be seen in every stage of change, from the first invasion to complete necrosis. This appearance is quite characteristic and most frequently observed in the skull.

The idea that mercury is responsible for these bone lesions should be received with a degree of caution. Evidence is abundant to show that untreated syphilis may present many bone lesions; on the other hand, it is shown that workers in mercury are no more liable to contract bone disease than other persons. Syphilitic necrosis may attack the maxillary bones usually beginning in the alveolar process of the superior.

First, the gums are red and swollen, gumma is formed and the teeth involved in the process are loosened. Ulceration soon takes place with a foul and offensive discharge. There is great difficulty of speech, mastication and deglutition. The teeth may be lost and a large portion of the bone destroyed. The hard palate is frequently involved, the disease beginning in a very insidiuous manner. A slight elevation without pain or redness or perforation may be the first sign of the disease. Fortunate for the patient only a small portion of the bone is lost; the soft parts quickly show a tendency to heal.

When proper stimulation is applied to the granulating edges the soft parts may be induced to repair the deformity, thus shutting off the nasal passage. This does away with the "nasal twang" in talking and prevents the passing of food and drink into the nasal passage. Again the result may not be so fortunate. The borders may be rapidly infiltrated and the necrosis may extend until the bones of the hard palate, the nasal bones and part of the maxilla are destroyed, producing frightful deformity. When the sequestrum has separated and the opening in the palate does not close surgical aid must be invoked, or if the damage is too great for the surgeon to repair the dentist must supply the deficiency by artificial means. Treatment must not be dropped for some time that there may be no return of the disease.

### HEREDITARY SYPHILIS,

Sometimes called congenital or infantile syphilis. According to statistics one-third of all the children born of syphilitic parents are dead when born, and 70 to 83 in every 100 children born of syphilitic parents die some time in infancy or childhood from no other cause than syphilis. The disease may appear as early as the third week, or it may be delayed until the twelfth week of life. This period is one of great danger to life. If the child lives the manifestations of disease may wholly disappear and from this time forward the child will seem to thrive. The danger of death diminishes as the child grows older. If there be no appearance of the disease until the child is six months old we may expect that he will reach manhood.

There are dangers at every turn in the pathway of life for the child who inherits syphilis. The organs of special sense—the eye and ear—are subject to inflammations which may lead to impaired sight and hearing. The course of inherited syphilis differs from the acquired in that it has no primary stage. It never begins with a local lesion and cannot be divided into stages. Many of the lesions are similar to those found in the acquired disease, are generally symmetrical but involve larger surfaces. The nasal symptoms, usually so late in acquired syphilis, are the first to appear in the inherited disease. Early ossification and changes in the bones are among the common lesions. Skin eruptions are also common, coming as a symmetrical erythema.

Evidences of hereditary taint will disappear under proper treatment by the time the child is sixteen years old, though some lesions have been observed long after this period if the youth be inclined to vicious habits. How much immunity inherited syphilis may exert upon the individual is hard to determine, though it is certain that it does not give full protection.

Syphilis is always transmitted as syphilis, though some writers think they can see in it scrofula, rickets and phthisis a parentage of syphilis. The duration of the disease depends much on the treatment and severity as well as the form which it may assume. Some children will present mild lesions for a few months, then become healthy again; others may have severe lesions lasting a short time, to be followed by numerous relapses. Late manifestions have not the degree of malignancy which belongs to earlier lesions.

Inherited syphilis is more chronic and varied in its course than the acquired. Lesions of the mucous membrane are numerous and extensive. There may be numerous cerebral and nervous symptoms, changes in the teeth and bones of the skull, which leave their marks for all time. How can the diagnosis of hereditary syphilis be made? While it may be important, our inquires must be pushed with caution, not to excite suspicion. Asking the mother pointed questions might unjustly arouse a suspicion in her mind that would poison her life. The father is usually the guilty person, and can be approached with far more freedom than the mother.

When the child has reached the proper age the permanent teeth may present symptomatic lesions of the disease. Early changes may be seen in the gums and teeth that are not truly symptomatic of syphilis. A case is reported by R. W. Parker in the *Times and Gazette* as follows:

The case came under his care when the child was three weeks old, but in spite of good treatment it lived to be only two months old. Corresponding to the place for two upper central incisors were two "gum-boils" the size of large peas; the rest of the gums were red and inflamed. When one of these "boils" was lanced a tooth dropped out.

Hutchinson, who has made extensive observations on syphilis in connection with the teeth, says:

"There are teeth, and they are seen not infrequently, so characteristic that I should feel sure in the absence of all other facts, or even in spite of them, that the possessor of such teeth must have inherited syphilis. Many cases there are where the changes are not great enough to warrant an opinion; they only lead to suspicion."

He has given the points of diagnosis so clearly that I might well give them to you. He says:

"We must not forget that the milk-teeth are never characteristically malformed. Nor ever neglect the rule that it is to the upper central incisors almost solely that we should look. The lower incisors may be malformed, but not characteristically, and this may be due to other causes than syphilis. The tooth-sac may suppurate and crown exfoliate before fairly cut. I have never witnessed the suppurative exfoliation of the upper incisors except in those who have syphilis. More common than exfoliation is caries of a peculiar form. The neck is attacked, eaten through and the crown drops off. We find these children from two to six years of age without teeth."

The uncommon symptoms he ranks as suspicious and not certain. Mistakes may easily be made in the permanent teeth. They may be dented or fluted as a result of early stomatitis. This condition may be more conspicuous than the signs of syphilis. He gives the following rules:

"All transverse markings (where they cross several teeth) are to be disregarded. All mere defects in enamel formation leading to pits or honeycombing, or irregular breakages, are to be disregarded. Foliated or wart-like projections on the teeth, with, of course, defects of enamel, although very suspicious, are untrustworthy. What we call peg-like teeth are also suspicious but not trustworthy. In looking at the test-teeth (the upper antral incisors), we must observe chiefly their size and edges."

The notch is more or less crescentic, and is accompanied by a rounding off of the angles. The tooth is almost always dwarfed both as regards its length and width. It is not flattened out as a normal incisor, but rounded and more or less pegged-shape. The enamel may be deficient in the middle of the notch. The teeth are often not properly placed, but incline towards each other or in opposite directions. They are seldom large enough to touch both their neighbors. The teeth are not imperfect in all cases of hereditary syphilis; on the contrary, they may be perfect. An ulcerating gumma of palate, which is almost conclusive proof of syphilis, has been seen when the teeth were perfect.

Mackenzie, of Baltimore, after a careful examination of one hundred and fifty infants, finds that 1st ulceration may take place in the palate, pharynx and naso-pharynx any time from the first week to the fourteenth year.

2. When eruptions are delayed the lesions of pharynx and palate are more common and persistent, being the first to draw attention to the disease.

3. Females are attacked more frequently than males.

4. Ulceration may occur at any point, but most common in the palate.

5. When seat of ulceration is posterior to the hard palate the naso-pharynx and nose are the parts invaded; when upon the palate it forms a way through the nasal cavity.

6. The next most common seats of ulceration in order of frequency are the fauces, naso-pharynx, post-pharyngeal wall, nasal fossa and septum, tongue and gums.

7. Ulceration of palate tends to necrosis of bone.

8. Tendency to necrosis exists at all times of life, but especially in early youth, when it is most destructive and less amenable to treatment.

All organs may be diseased in inherited syphilis. There may also be disease of the bones, joints and loss of hair. When the eyebrows are missing at two or three months syphilis may be suspected. When syphilis is suspected, study well your case before arriving at a diagnosis. When in doubt, recommend treatment for syphilis and carefully watch the effect.

